# Cyclooxygenase activity is important for efficient replication of mouse hepatitis virus at an early stage of infection

**DOI:** 10.1186/1743-422X-4-55

**Published:** 2007-06-07

**Authors:** Matthijs Raaben, Alexandra WC Einerhand, Lucas JA Taminiau, Michel van Houdt, Janneke Bouma, Rolien H Raatgeep, Hans A Büller, Cornelis AM de Haan, John WA Rossen

**Affiliations:** 1 Virology Division, Department of Infectious Diseases & Immunology, Faculty of Veterinary Medicine, Utrecht University, Utrecht, The Netherlands; 2 Laboratory of Pediatrics, Erasmus MC- Sophia Children's Hospital, Rotterdam, The Netherlands; 3 Department of Virology, Eijkman-Winkler Institute, University Medical Centre Utrecht, Utrecht, The Netherlands; 4 Laboratory of Medical Microbiology and Immunology, St. Elisabeth Hospital, Tilburg, The Netherlands

## Abstract

Cyclooxygenases (COXs) play a significant role in many different viral infections with respect to replication and pathogenesis. Here we investigated the role of COXs in the mouse hepatitis coronavirus (MHV) infection cycle. Blocking COX activity by different inhibitors or by RNA interference affected MHV infection in different cells. The COX inhibitors reduced MHV infection at a post-binding step, but early in the replication cycle. Both viral RNA and viral protein synthesis were affected with subsequent loss of progeny virus production. Thus, COX activity appears to be required for efficient MHV replication, providing a potential target for anti-coronaviral therapy.

## Background

Virus infections often cause acute inflammatory responses, which are mediated by several cellular effectors and soluble factors. Although these responses have an important protective role, they may also have deleterious effects on the host. The balance between these protective and deleterious effects may ultimately determine the course of disease after viral infection. Prostaglandins (PGs) are important regulators of this inflammatory reaction. They are synthesized by cyclooxygenases (COXs), converting arachidonic acid into PGH_2_, which can then be isomerized to generate different biologically active forms of PGs. There are three known isoforms of COXs, with COX-1 and COX-2 being the best characterized. COX-1 is expressed in various cell types and PGs produced by COX-1 are predominantly involved in the regulation of various homeostatic processes [1]. COX-2 is an immediate early response gene, which upon induction generates mainly hyperalgesic and proinflammatory PGs at sites of inflammation [2,3]. PGs from the E series, such as PGE_2_, also exhibit immunomodulatory activities, preventing hyperactivation of the innate cellular immunity [4]. Furthermore, they can inhibit the secretion of gamma interferon, a cytokine with antiviral activity [5]. A direct role for COXs and PGs in controlling viral replication has been described for a wide range of virus infections, but their actions appear to be dependent on both the virus and cell type [6]. For instance, COXs and/or PGs are required for efficient replication of herpesviruses [7-13], bovine leukemia virus [14], and rotavirus [15]. In case of human cytomegalovirus, human T-lymphotropic virus type 1, and human immunodeficiency virus type-1 PGE_2 _has been shown to stimulate virus replication by activating viral promotors [16-18]. On the other hand, COXs/PGs negatively affect adenovirus replication, as well as replication of human immunodeficiency virus type 1 in macrophages [19,20]. The mechanisms by which COXs and PGs regulate viral replication are largely unclear.

Coronaviruses (CoVs) constitute a family of enveloped, positive-stranded RNA viruses. They are known pathogens in the veterinary field, causing severe diseases in several domestic species [21]. Recently, their relevance has increased considerably with the discovery of several new human CoVs (HCoVs) such as the severe acute respiratory syndrome (SARS)-CoV [22], HCoV-NL63 [23], and HCoV-HKU1 [24]. The role of COXs during CoV infection and pathogenesis is not well understood. MHV strain 3, which causes fulminant hepatitis, was shown to induce the synthesis of PGE_2 _in macrophages [25]. However, the exogenous administration of PGE_2 _could completely prevent the development of hepatic necrosis [26]. More recently, two structural proteins from the SARS-CoV were shown to induce the expression of COX-2 *in vitro *[27-29], whereas elevated levels of PGE_2 _were found in the blood of SARS-CoV-infected individuals [30], suggesting a role for COXs and PGs in CoV pathogenesis. However, the requirement for COX activity for CoV replication remains unexplored.

## Results

**Figure 1:**
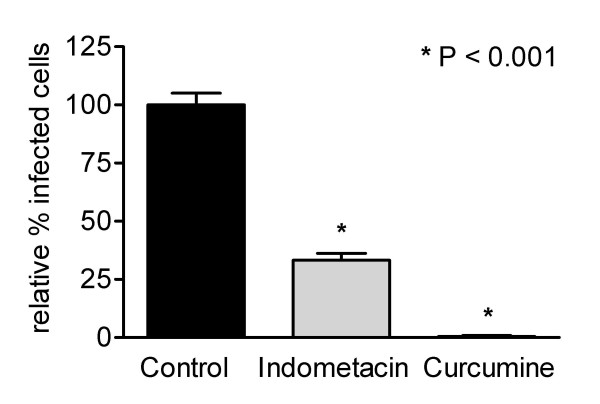
**COX inhibitors are negatively affecting MHV infection**. (A) Caco-MHVR cells were incubated with culture medium (containing a concentration of DMSO similar to that present in the inhibitor solutions), 20 μM indomethacin, or 30 μM curcumine 1 h prior to inoculation with MHV-A59 (m.o.i = 0.01). The cells were maintained in the presence of the inhibitors until they were fixed at 6 h p.i. Infected cells were detected by an indirect IFA using an anti-MHV serum and Texas Red conjugated secondary antibodies. Fluorescence was viewed with a Nikon Eclipse E800 microscope. The numbers of MHV-infected cells in the drug-treated cells are presented as a percentage of the average number of infected cells in the mock-treated (control) cell cultures. Data are presented as mean ± standard error of mean (n = 6). For statistical analysis a one-way ANOVA with the Tukey-Kramer test was performed using GraphPad Prism version 3.00 for Windows (GraphPad Software). In all tests, *P *< 0.05 was considered statistically significant.

In the present study we investigated the role of COXs in the MHV replication cycle. To this end, Caco-2 cells, stably expressing the MHV receptor glycoprotein (Caco-MHVR) [31], were infected with MHV strain A59 (MHV-A59) at a multiplicity of infection (m.o.i.) of 0.01 in the presence or absence of the COX-1 and COX-2 inhibitors indomethacin and curcumine. The cells were incubated 1 h prior to infection with the inhibitors, and were maintained in the presence of the inhibitors from 30 minutes post infection (p.i.). Cells were fixed at 6 h p.i. with ice-cold methanol, and the number of MHV-infected cells were determined by an indirect immunofluorescence assay (IFA) using anti-MHV antibodies [32]. Possible cytotoxic effects of the inhibitors and their solvents were tested, using cell proliferation reagent WST-1 and lactate dehydrogenase cytotoxicity detection kit (Roche Diagnostics) assays according to the manufacturer's protocol. All inhibitors were used at concentrations that were not toxic to the cells. In the presence of 20 μM indomethacin, MHV infection was reduced by 57%, while curcumine reduced infection by 95% at a concentration of 30 μM (Figure 1). Both drugs affected MHV infection in a concentration-dependent manner (data not shown).

**Figure 2:**
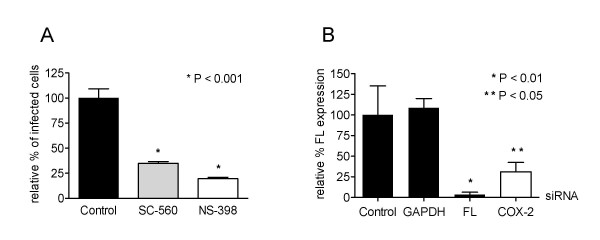
**Blocking COX-1 or COX-2 activity by specific inhibitors, or by siRNAs targeting COX-2 mRNA reduce MHV infection**. (A) Caco-MHVR cells were incubated with COX-1 (SC-560; 1 μM) or COX-2 (NS-398; 0.055 μM) inhibitor 1 h prior to inoculation with MHV-A59 (m.o.i. = 0.01) and were maintained in the presence of the inhibitors until they were fixed. The numbers of MHV-infected cells were determined with an indirect IFA and are presented as described in the legend of Figure 1. (B) HeLa cells were transfected with 10 nM siRNAs, targeting the indicated transcripts, 72 h prior to inoculation with MHV-FLSrec. Cell viability was measured for 30 minutes at 6 h p.i. using a WST-1 assay as described previously [37], after which the intracellular luciferase levels were determined as relative light units (RLU). Luciferase levels in siRNA-transfected cells are expressed as a percentage of the levels in the mock-transfected (control) cells and were corrected for the percentage of viable cells (n = 3).

Next, we determined the role of the different COX isoforms. The ability of specific COX-1 and COX-2 inhibitors to reduce MHV infection was determined in a similar way as described above. Both SC-560 and NS-398, which inhibit COX-1 and COX-2, respectively, reduced MHV infection by 65–75% at concentrations that were non-toxic to the cells (1 μM and 0.055 μM respectively) (Figure 2A). Apparently, the activity of both enzymes is required for efficient MHV replication in Caco-MHVR cells. RNA interference technology was applied to confirm the observation that COX-2 activity is important for MHV replication. Parallel cultures of HeLa cells were transfected with siRNAs (purchased from Dharmacon, Inc.) targeting COX-2, firefly luciferase (FL) (positive control) or GAPDH (specificity control) transcripts for degradation. They were infected at 72 h posttransfection with MHV-FLSrec [33], a recombinant MHV expressing the FL reporter gene, the level of which is a reliable measure for MHV replication [34]. Silencing of GAPDH, a cellular housekeeping gene, did not affect FL expression compared to mock-transfected (control) cells (Figure 2B). However, HeLa cells transfected with siRNAs targeting the FL or COX-2 transcripts showed a reduction in FL expression of more than 90% and 65%, respectively. A taqman reverse transcription (RT)-PCR targeting COX-2 mRNA revealed that in cells treated with COX-2 siRNAs, COX-2 mRNA levels were decreased with more than 70% compared to control cells (data not shown). Therefore, these data show the requirement of COX-2 activity for efficient MHV replication.

**Figure 3:**
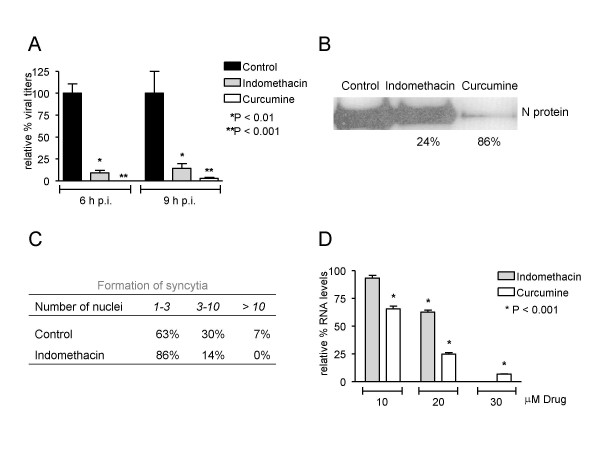
**Indomethacin and curcumine inhibit MHV replication at the level of RNA synthesis**. Caco-MHVR cells were incubated with and maintained in culture medium containing DMSO, 20 μM indomethacin, or 30 μM curcumine as described in figure legend 1. After 1 h, the cells were inoculated with MHV-A59 (m.o.i. = 1). At 6 and 9 h p.i., supernatants were collected and cells were harvested to isolate infectious viral particles, proteins and total RNA. (A) Caco-MHVR cells were inoculated with serial dilutions of combined supernatants and cleared cell homogenates from mock-treated (black bars), indomethacin-treated (grey bars) and curcumine-treated (white bars) cultures collected at 6 and 9 h p.i. The amount of ffu in the samples was determined with an indirect IFA as described in the legend of Figure 1 (n = 3). (B) Protein samples were analyzed on a SDS-15% polyacrylamide gel followed by Western blotting using the polyclonal anti-MHV serum. The N protein levels and the percentage of reduction (normalized for β-tubulin expression (data not shown)) in drug-treated cells compared to mock-treated cells are indicated. (C) The size of the observed syncytia was measured by counting the number of nuclei per syncytium of MHV-infected cells in the absence or presence of 20 μM indomethacin. (D) The expression levels of the N gene of MHV were determined by Taqman RT-PCR using primers 2915 (5'-GCCTCGCCAAAAGAGGACT-3') and 2916 (5'-GGGCCTCTCTTTCCAAAACAC-3') and a dual labeled probe (5'-6-FAM-CAAACAAGCAGTGCCCAGTGCAGC-TAMRA-3'). The relative amount of viral RNA in the drug-treated cells was expressed as a percentage of the average amount of viral RNA in the mock-treated cells.

To determine which step of the MHV replication cycle was affected by the COX inhibitors, the production of infectious particles, of viral protein and of viral RNA was analyzed. For this purpose, Caco-MHVR cells were inoculated with MHV-A59 (m.o.i. = 1) in the presence or absence of indomethacin or curcumine. The amount of infectious viral progeny present in cells and culture media was monitored by determining the number of fluorescent focus-forming units (ffu) at different time points p.i. Inhibition of COX activity by curcumine and indomethacin resulted in a significant decrease in the yield of infectious viral progeny by more than 95% and 85%, respectively (Figure 3A). In addition, the amount of N protein present in cell lysates was analyzed by Western blotting using a polyclonal anti-MHV serum. N protein expression levels were markedly reduced by curcumine (86% reduction), and to a lesser extent by indomethacin (24%) (Figure 3B). Consistent with these results, much smaller syncytia were observed after infection of Caco-MHVR cells in the presence of the COX inhibitors (Figure 3C). Reduced expression levels of the MHV S protein, which is responsible for cell-cell fusion in MHV-infected cells [35] are likely to explain the lack of syncytium formation after COXs inhibition. Finally, viral RNA synthesis was analyzed in the presence of COX inhibitors. At 6 h p.i., total RNA was isolated and viral RNA synthesis was monitored by Taqman RT-PCR using a probe and primers that detect the N gene (details in legend Figure 3). Indomethacin and curcumine both inhibited viral RNA synthesis in a dose-dependent manner (Figure 3D). These results indicate that the COX inhibitors interfere with viral RNA and protein synthesis and consequently affect the production of infectious particles. In agreement with our findings, a recent study described the potent antiviral effect of indomethacin on SARS and canine coronavirus (CCoV) replication [36].

**Figure 4:**
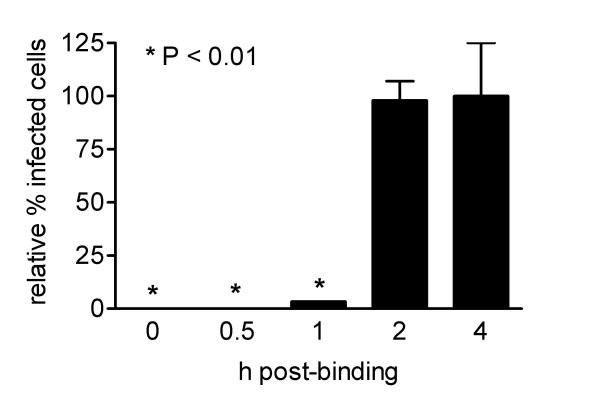
**COX inhibition affects MHV infection at a post-binding step**. Caco-MHVR cells were inoculated with MHV-A59 (m.o.i. = 0.01) at 4°C for 2 h. Subsequently, cells were placed at 37°C and 20 μM indomethacin was added to the culture medium immediately (t = 0 h post binding) or at the indicated times. Cells were maintained in culture medium containing indomethacin until they were fixed at 6 h post-binding. The numbers of infected cells are presented as described in the legend of Figure 1 (n = 3).

To study the kinetics of inhibition of MHV replication in more detail Caco-MHVR cells were inoculated with MHV-A59 (m.o.i. = 0.01) for 2 h at 4°C to allow binding of the virus to the cells without entry. After removing any unbound viral particles, the cells were placed at 37°C to induce virus entry and 20 μM indomethacin was added at the time points indicated (Figure 4). MHV infection was significantly reduced, as measured by the indirect IFA described above, if indomethacin was added up to 1 h after the cells were placed at 37°C. The maximum inhibitory effect was obtained when indomethacin was added immediately after the cells were placed at 37°C. No significant inhibition of the infection was observed if indomethacin was added 2 h after the cells were placed at 37°C. This result demonstrates that COX activity plays an important role early in the virus infection cycle, at a post-binding step. Thus, COX activity might either be required for efficient entry or for an initial step in RNA replication. Similarly, rotavirus replication was also negatively affected by the addition of COX inhibitors early, but not late in the infection cycle [15]. In conclusion, our results clearly show that COX activity is required for efficient virus replication *in vitro *early during MHV infection. These findings may offer new possibilities for anti-CoV therapy.

## Competing interests

The author(s) declare that they have no competing interests.

## Authors' contributions

MR, LJAT, MvH, JB, JWAR, and RR conducted all the experiments. MR wrote the manuscript. AWCE, HAB, CAMdeH, and JWAR coordinated the research efforts and assisted with writing the manuscript. All authors read and approved the final manuscript.
